# Rare variants in the splicing regulatory elements of EXOC3L4 are associated with brain glucose metabolism in Alzheimer’s disease

**DOI:** 10.1186/s12920-018-0390-6

**Published:** 2018-09-14

**Authors:** Jason E. Miller, Manu K. Shivakumar, Younghee Lee, Seonggyun Han, Emrin Horgousluoglu, Shannon L. Risacher, Andrew J. Saykin, Kwangsik Nho, Dokyoon Kim, for the Alzheimer’s Disease Neuroimaging Initiative

**Affiliations:** 1 Biomedical and Translational Informatics Institute, Geisinger Health System, Danville, PA USA; 2 Department of Biomedical Informatics, University of Utah School of Medicine, Salt Lake City, UT 84106 USA; 3 Department of Radiology and Imaging Sciences, Indiana University School of Medicine, Indianapolis, IN USA; 4 Huck Institute of the Life Sciences, Pennsylvania State University, University Park, PA USA; 5 Present Address: Department of Genetics, Institute for Biomedical Informatics, Perelman School of Medicine, University of Pennsylvania, Philadelphia, PA USA

**Keywords:** Alternative splicing, Imaging genomics, Alzheimer’s disease, Whole genome sequencing, Rare variants

## Abstract

**Background:**

Alzheimer’s disease (AD) is one of the most common neurodegenerative diseases that causes problems related to brain function. To some extent it is understood on a molecular level how AD arises, however there are a lack of biomarkers that can be used for early diagnosis. Two popular methods to identify AD-related biomarkers use genetics and neuroimaging. Genes and neuroimaging phenotypes have provided some insights as to the potential for AD biomarkers. While the field of imaging-genomics has identified genetic features associated with structural and functional neuroimaging phenotypes, it remains unclear how variants that affect splicing could be important for understanding the genetic etiology of AD.

**Methods:**

In this study, rare variants (minor allele frequency < 0.01) in splicing regulatory element (SRE) loci from whole genome sequencing (WGS) in the Alzheimer’s Disease Neuroimaging Initiative (ADNI) cohort, were used to identify genes that are associated with global brain cortical glucose metabolism in AD measured by FDG PET-scans. Gene-based associated analyses of rare variants were performed using the program BioBin and the optimal Sequence Kernel Association Test (SKAT-O).

**Results:**

The gene, EXOC3L4, was identified as significantly associated with global cortical glucose metabolism (FDR (false discovery rate) corrected *p* < 0.05) using SRE coding variants only. Three loci that may affect splicing within EXOC3L4 contribute to the association.

**Conclusion:**

Based on sequence homology, EXOC3L4 is likely a part of the exocyst complex. Our results suggest the possibility that variants which affect proper splicing of EXOC3L4 via SREs may impact vesicle transport, giving rise to AD related phenotypes. Overall, by utilizing WGS and functional neuroimaging we have identified a gene significantly associated with an AD related endophenotype, potentially through a mechanism that involves splicing.

## Background

Late-onset Alzheimer’s disease (LOAD) is a progressive common neurodegenerative disorder that causes problems with memory, thinking, and behavior and pathologically characterized by the presence of amyloid deposition and neurofibrillary tangles in the brain [1, 2]. 5.5 million Americans are estimated to have AD in 2017 and the number of Americans with AD is rapidly increasing because of the growing number of older adults [1]. Currently, there is no available cure for AD. As a result, without earlier diagnosis and early disease-modifying intervention, the total number of individuals with AD is predicted to quadruple by 2050, causing a great economic and social burden [1]. Furthermore, a biomarker for early diagnosis could benefit clinical trials for AD by precisely classifying prognosis, stage, and determining a clinical endpoint [3]. Thus, AD related research is increasingly important, especially as it relates to early diagnosis.

Genetic variation may play an essential role in AD pathogenesis [4]. Recently, a large-scale genome-wide association study (GWAS) identified and validated more than 22 susceptibility genes for LOAD [5]. After the success of GWAS for common SNPs, large-scale whole exome and genome sequencing studies have successfully identified several rare risk variants for LOAD [6–9]. Recently, the genetics of AD has been investigated in the context of imaging data. By combining information from genetic architecture, functional neuroimaging, and multi-omics data, genetic variation associated with AD-related imaging biomarkers have been identified and thus the potential influence of genetic variation on brain structure and function related to AD pathophysiology [10, 11]. Furthermore, imaging endophenotypes can substantially increase statistical detection power of genetic association analysis through the use of quantitative traits as phenotypes [12].

Rare and low-frequency variants play an important role in the heritability of disease. However, the spurious nature of rare variants makes them difficult to run an association test. With so few occurrences of the variant at a given loci most tests will be underpowered [13]. In order to overcome this problem, variants can be grouped together by prior biological knowledge, such as genes, conserved loci, and pathways [14–16]. This strategy will accumulate effects of rare variants within a knowledge-driven region and reduce the number of statistical tests, thereby increasing the power to detect an association. Additionally, focusing on specific types of variants, such as those that lead to non-synonymous changes can also reduce the multiple testing burden and provide a potential explanation for the gene associated with the phenotype [17]. An attractive category of variant for studying AD are those that impact splicing.

Alternative splicing (AS) is an important gene regulatory mechanism underlying neurological function and development [18]. While motifs along splice junctions have been well studies, the effect of genetic variants in splicing regulatory elements (SREs) is less understood in the context of AD. There are multiple types of SREs that can impact splicing in different ways including intronic splicing enhancers (ISE), intronic splicing silencers (ISS), exonic splicing enhancers (ESE), and exonic splicing silencers (ESS) [19]. However, the significance of SRE in relation to AD related phenotypes remains unknown. In this study, an imaging genomics approach was taken to investigate AD by identifying rare variants within SREs associated with a neuroimaging phenotype using ADNI data. The Alzheimer’s Disease Neuroimaging Initiative (ADNI) has provided publically available whole-genome sequencing (WGS) data, along with imaging phenotypes. BioBin, an open-source program that was developed to group or bin the variants using information from multiple databases, was employed to bin rare variants from ADNI WGS data [14]. Gene-based analyses were performed using the optimal Sequence Kernel Association Test (SKAT-O), which maximizes power by adaptively using the data to optimally combine the burden test and dispersion test.

## Methods

### Study sample

All whole-genome sequencing (WGS) and imaging data came from the Alzheimer’s Disease Neuroimaging Initiative (ADNI) cohort. The cohort used here was made up of participants with cognitive normal (CN), early mild cognitive impairment (EMCI), late MCI (LMCI), and AD. The demographic data, along with sequencing and imaging data were downloaded from the ADNI data repository (http://www.loni.usc.edu/ADNI/). All participants provided written informed consent and study protocols were approved by participating sites’ Institutional Review Board. WGS was performed using blood-derived genomic DNA samples from ADNI participants. Sequencing was performed using 100 bp paired-end reads on the Illumina HiSeq2000 platform (www.illumina.com). As previously described using Broad GATK and BWA-mem, reads were mapped and aligned to the human genome (build 37), then variants were called [8, 20].

### Neuroimaging analysis

Pre-processed [^18^F] FDG PET scans were downloaded from the LONI (http://loni.usc.edu). As previously described in detail, these FDG scans (Co-registered, Averaged, Standardized Image and Voxel Size, Uniform Resolution) were already averaged, aligned to a standard space, re-sampled to a standard image and voxel size (2 × 2 × 2 mm), smoothed to a uniform resolution, and intensity normalized [21]. The pre-processed images were aligned to each individual’s MRI scan at the same visit and normalized to MNI space using SPM8 as previously described [22]. The intensity of the resulting scans was re-scaled to a pons reference region and then the final [^18^F] FDG standardized uptake value ratio (SUVR) images were created. A global cortical glucose metabolism measured from [^18^F] FDG-PET scans was used as an AD-related quantitative endophenotype with age at baseline and sex as covariates.

### Variant annotation

The VCF file containing 695 non-Hispanic Caucasian participants with imaging phenotype, covariates and genomic data was annotated using the variant effect predictor (VEP) package [23]. The variant_effect_predictor.pl script was applied to the VCF file using cache, refseq, and pick flags. Variants were then selected if they were also annotated with an SRE element. Sequences and organization of SREs in humans were identified using previously developed method [19, 24] which required the use of dbSNP version 137 and hg19 reference genome [25, 26]. In brief, this method predicts hexamer motifs associated with the following types of SREs associated with exon skipping events: intronic splicing enhancer (ISE), exonic splicing enhancer (ESE), and exonic splicing silencer (ESS). The program *twoBitToFa* was used to find the genome sequences surrounding the SNPs of interest with hg19 reference [27]. 11-mer sequences were interrogated surrounding the SNPs (5 bp on each side) using the hexamer motifs. While ESE and ESS were coding SNPs, the ISE were defined as intronic SNPs. Using the published methods [19], an SRE was counted if there was a match between a hexamer motif and any part of the 11-mer, and associated with exon skipping. ISE SNPs were included based on the exons bordering the intron in which the SNP was located.

### Variant binning, association test, and analysis

BioBin was used to group rare variants by genic region (minor allele frequency (MAF) < 0.01). BioBin uses gene annotations from LOKI (the library of knowledge integration), which contains a number of widely used publically available databases such as NCBI Entrez, UCSC Genome Browser, Kyoto Encyclopedia of Genes and Genomes (KEGG), Reactome, Genome Ontology (GO) and others. Association tests were performed using SKAT-O [28], adjusting for age and sex. The minimum bin size included in the association test was five variants across samples. The bins were tested for an association with global brain cortical glucose metabolism measured by FDG PET scans (often referred to as the “imaging phenotype”). For both annotation and BioBin, the GRCh37 assembly was specified. Finally, the false discovery rate for BioBin output *p*-values was then calculated in R using the p.adjust function, using the “FDR” method. Variant effect analysis using PROVEAN and SIFT was performed online at http://provean.jcvi.org [29–31]. The UCSC genome browser was used to visualize the *ECO3L4* gene along with splicing isoforms and protein domains [25].

## Results

**Fig. 1:**
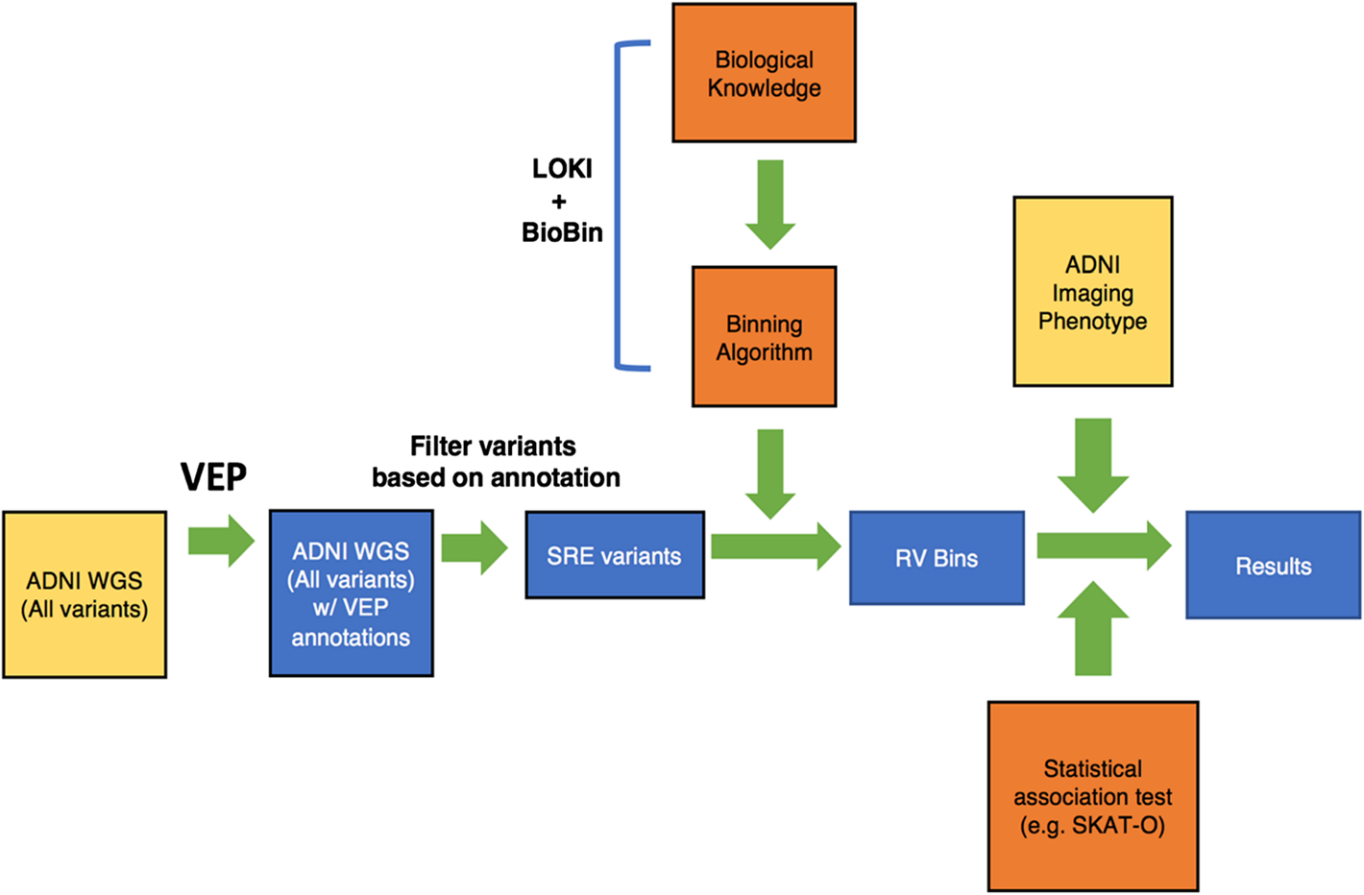
Workflow describing rare SRE variant association test using imaging phenotype data. Diagram of how rare variants (RV) from whole-genome sequencing (WGS) data were tested for an association with ADNI imaging data. WGS variants were annotated with VEP then filtered for those that reside in SRE loci (i.e., ESE, ESS, and ISE). Variants were then binned into genes using annotations from LOKI. SKAT-O was then used to test genes for an association with the ADNI imaging endophenotype

**Fig. 2:**
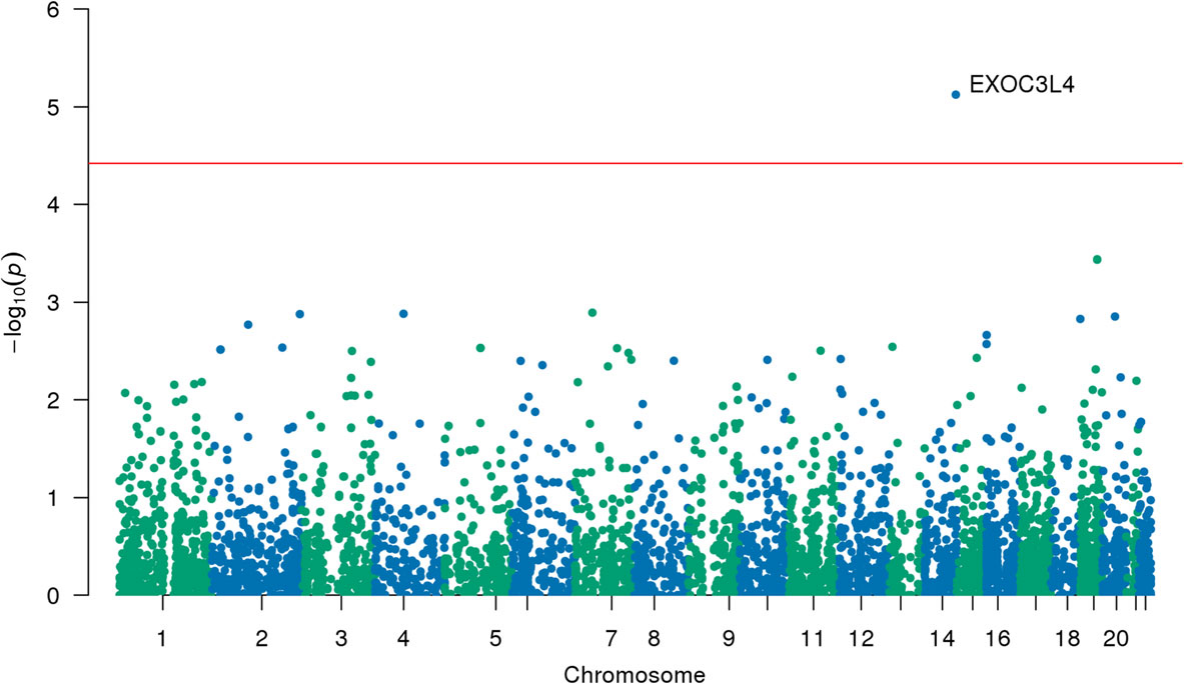
Manhattan plot of *p*-values from association between genes and the imaging phenotype using SRE coding variants. Manhattan plot which shows the results from the association test between the imaging phenotype and each gene tested using SKAT-O. Only variants that fell into SRE coding loci were used. The blue and red lines represent 0.05 *p*-value and 0.05 FDR cutoffs, respectively

First, variants from the ADNI WGS study were selected that were located in the SRE coding and/or ISE loci (Fig. 1). Next BioBin was employed to bin variants with minor allele frequency (MAF) less than 0.01 into their respective genes. SKAT-O was used to test if these rare variants in each gene were associated with the phenotype derived from FDG PET scans from ADNI. These associations were adjusted for covariates such as age and sex to reduce the effect of confounding variables (Table 1). The *p*-values were adjusted for multiple testing, and genes with a false discovery rate (FDR) less than 0.05 were considered significant, while those with FDR < 0.1 were suggestive of statistical significance. Using ISE variants, there were no genes that had a significant association with the imaging phenotype (Table 2). However, using SRE coding variants (i.e., ESE and ESS), *EXOC3L4* was identified as having a genome-wide significant (FDR < 5%) association with the imaging phenotype (Table 3 and Fig. 2). PROVEAN and SIFT predictions for variants in *EXOC3L4* were considered neutral and tolerated, respectively, which suggests SRE annotations offer novel insight into the consequence of rare variants. Also, the sequence homology between *EXOC3L4* and exocyst complex components suggests SRE sites are within the Sec6 domain, indicating these splicing elements could have a functional impact on the protein (uniprot.org). Alternatively, when combining both sets of variants there were several genes which were only suggestive of having a significant (FDR < 10%) association with the imaging phenotype (Table 4). In summary, SRE coding annotations provided increased power to detect *EXOC3L4* as having an association with the imaging phenotype.

**Table 1:** Summary statistics of variables used as covariates in association study

Demographics and Covariates	Values (*N* = 695)
Sex (M/F)	391/304
Age in years (Mean/Std)	72.95 (+/− 7.05)

**Table 2:** Top 5 genes associated with imaging phenotype using ISE variants only

Gene	Unique Loci	Variants across cohort	SKAT-O *p*-value	FDR corrected *p*-value
TNFAIP2	7	10	2.47E-05	0.123
STK35	56	148	3.09E-05	0.123
PWRN1	34	101	4.15E-05	0.123
EXOC3L4	8	21	6.11E-05	0.123
TMEM182	23	67	6.22E-05	0.123

**Table 3:** EXOC3L4 gene is associated with imaging phenotype using ESE/ESS variants only

Gene	Unique Loci	Variants across cohort	SKAT-O *p*-value	FDR corrected *p*-value
EXOC3L4	4	16	7.48E-06	0.038

**Table 4:** Top 5 genes associated with imaging phenotype using ESE/ESS and ISE variants

Gene	Unique Loci	Variants across cohort	SKAT-O *p*-value	FDR corrected *p*-value
TNFAIP2	8	14	1.20E-05	0.094
EXOC3L4	10	35	1.99E-05	0.094
STK35	56	148	3.09E-05	0.094
STEAP4	7	13	3.23E-05	0.094
PWRN1	34	101	4.15E-05	0.097

**Fig. 3:**
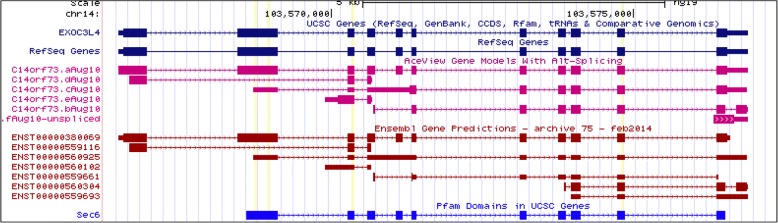
Screen shot of EXOC3L4 in the UCSC genome browser. Exons are marked by thick square blocks while the thin lines with hash marks represent intronic regions

To investigate the association with *EXOC3L4* further, each of the four loci in *EXOC3L4* with rare variants were interrogated to define their contribution to the association. This analysis was performed by removing each SNP individually then rerunning the association test to retrieve a *p*-value for only *EXOC3L4* (Table 5). After removing SNPs rs10142287, rs9324055, or rs148718670, *EXOC3L4* had a *p*-value that was less significant compared to the original *p*-value of *EXOC3L4* with 4 variants. These effects are unlikely to be caused purely by the number of variants at each locus removed, as only one or three variants were removed, suggesting there is something specific about the loci which leads to the association with the phenotype. As shown in the UCSC genome browser (Fig. 3), there is evidence that alternative splicing of *EXOC3L4* can lead to the existence of a transcript that skips the second exon which harbors two SNPs within ESE sites, rs10142287 and rs9324055. The skipped exon is part of a region encoding the Sec6 domain (Fig. 3). These results help explain why variability in SRE sites of *EXOC3L4* could impact the phenotype through a mechanism involving AS. On the other hand, removing the rs117708804 SNP resulted in an increase in significance as illustrated by the lower *p*-value, suggesting that *EXOC3L4* can absorb variation at this locus, and that variants here may be spurious or not important for the context of this association.

**Table 5:** Characterization of EXOC3L4 rare variant loci

rsID	Consequence	*p*-value^a^	SRE type	Variants across cohort
rs117708804	missense	4.32E-07	ESE, ESS	11
EXOC3L4		7.48E-06^b^		16
rs10142287	synonymous	1.58E-04	ESE	1
rs9324055	missense	1.59E-04	ESE	1
rs148718670	missense	1.68E-04	ESE	3

^a^SKAT-O p-value results after removing the variant from EXOC3L4

^b^SKAT-O *p*-value result using all variants from EXOC3L4

## Discussion

Although very little is known about *EXOC3L4* or its orthologues, BLAST search results using its amino acid sequence suggests it is likely to be an exocyst complex component (uniprot.org). This information lends itself to a number of possible models for how *EXOC3L4* may be involved in AD. In mammals, the exocyst complex is an eight-subunit complex that is ubiquitously expressed [32]. The exocyst proteins are important for vesicle trafficking along with SNARE proteins [33, 34]. It has been suggested that SNARE proteins are important for glucose uptake in the context of proper neuronal function [35]. And the imaging phenotype from this study, FDG PET-scans, quantifies global brain cortical glucose metabolism in AD. Additionally, vesicle transport is used for lysosomal transport, such as seen in autophagy [36]. AD is defined by the accumulation of proteins like amyloid plaques, which can be removed via the autophagy-lysosome pathway [37]. There is evidence that defects in this autophagy process can lead to AD [38]. Thus, our results suggest a model where variants in *EXOC3L4* that are located in SRE coding loci may alter the function of the protein through exon skipping, which may inhibit proper vesicle transport. Moreover, these variants may lead to AD related phenotypes.

Evidence suggests the exocyst plays important roles in embryogenesis, neuronal cell polarity, and cell motility [32]. *EXOC3L4* shares high sequence similarity with M-seq (also known as *TNFaip2*), a protein that shares structural similarity to *Sec6* (uniprot.org). It has been suggested that *TNFaip2* has a role in filopedia development in neurons [32]. Thus, if *EXOC3L4* does not carry out its function through interactions with the exocyst complex, there is evidence that exocyst-like proteins are important for neuronal cell function through an alternative mechanism.

One limitation of this study is the samples size. *TNFAIP2* was only suggestive of statistical significance, however it is another protein that is functionally relevant to SNARE proteins and thus vesicle transport (uniprot.org). Since only few samples contained the rare variants, it will be important for this study to be replicated in another independent cohort. Additionally, since these are associations it will be important to perform follow-up experiments to identify a causal link between *EXOC3L4* and AD. None the less, these results suggest more genes that contain rare variants in SRE loci, and are important for proper exocytosis and autophagy, are likely to be identified in studies with increased sample size. In summary, annotating variants as SRE provides novel insight as to how rare variants may be useful when finding an association between an imaging phenotype and genetic variants in the context of AD.

## Conclusions

In this study, we set out to find associations between a neuroimaging phenotype and rare SRE variants from WGS in AD. While it is common to perform a gene-wise association test, we hypothesized that the power of this study could be increased by focusing on functionally relevant loci such as those that impact splicing. Furthermore, associations with annotated regions can lead to easier interpretation afterward. Thus, rare variants that fell into SRE loci from WGS from the ADNI cohort were collapsed into genes using BioBin. These rare variants were used in an association test for an imaging phenotype and *EXOC3L4* was identified as having a statistically significant association. And while intronic elements did not detect a statistically significant association, exonic splicing elements did. In summary, utilizing prior biological knowledge in the form of splicing elements serves as an important means to identify genotype-phenotype relationships with respect to imaging data and AD.

## Abbreviations

FDG: Fluorodeoxyglucose
LOAD: Late-onset Alzheimer’s Disease
WGS: Whole-genome sequencing
